# MKRN3 and KISS1R mutations in precocious and early puberty

**DOI:** 10.1186/s13052-020-0808-6

**Published:** 2020-03-30

**Authors:** Sara Pagani, Valeria Calcaterra, Gloria Acquafredda, Chiara Montalbano, Elena Bozzola, Pietro Ferrara, Manuela Gasparri, Alberto Villani, Mauro Bozzola

**Affiliations:** 1 Department of Internal Medicine and Therapeutics, Pediatrics and Adolescent Care Unit, University of Pavia, Via Aselli 43, 27100 Pavia, Italy; 2 Immunology and Transplantation Laboratory, Pediatric Hematology and Oncology, Fondazione IRCCS San Matteo, Pavia, Italy; 3 Department of Pediatrics, Bambino Gesù Children’s Hospital, Rome, Italy; 4 Institute of Pediatrics, Catholic University, Rome, Italy; 5 Department of Pediatrics, San Paolo Hospital, Milan, Italy

**Keywords:** Early onset of puberty, Precocious puberty, Genetic variations, MKRN3 and KISS1R genes, Growth factors

## Abstract

**Background:**

Pubertal timing is known to be influenced by interactions among various genetic, nutritional, environmental and socio-economic factors, although the ultimate mechanisms underlying the increase in pulsatile GnRH secretion at puberty have yet to be fully elucidated. The aim of our research was to verify the role of *KISSR1* (previously named *GPR54*) and *MKRN3* genes on pubertal timing.

**Methods:**

We analyzed the DNA sequence of these genes in 13 girls affected by central precocious puberty (CPP) who showed onset of puberty before 8 years of age, and in 6 girls affected by early puberty (EP) between 8 and 10 years of age.

**Results:**

Direct sequencing of the *KISS1R* (GPR54) gene revealed two SNPs. One SNP is a missense variant (rs 350,132) that has been previously reported in connection to CPP in Korean girls. The other variant that we found in the *GPR54* gene (rs764046557) was a missense SNP located in exon 5 at position 209 of the aminoacid. We identified this variant in only one CPP patient. Automatic sequencing of *MKRN3* in all patients revealed three variants in eight subjects. In 6 out of 19 (31.5%) patients (3/13 CPP patients and 3/6 EP patients) we found the synonymous variant c.663C > T (rs2239669). Another synonymous variant (rs140467331) was found in one of our CPP patients, as well as one missense variant (rs760981395) in another CPP patient.

**Conclusion:**

In conclusion, we identified sequence variations of the *KISS1R* and *MKRN3* genes, two of the most frequent genetic causes of ICPP. Our results suggest that these variants might be inducible factors in the pathogenesis of CPP.

## Introduction

Puberty is a complex biological process of sexual development, controlled at hypothalamic level by activation of pulsatile gonadotropin-releasing hormone (GnRH) secretion, which stimulates hormonal cascade and gonadal activation [1].

Pubertal timing is known to be influenced by interactions among various genetic, nutritional, environmental and socio-economic factors, although the ultimate mechanisms underlying the increase in pulsatile GnRH secretion at puberty have yet to be fully elucidated [2].

Central precocious puberty (CPP) is clinically identified by the development of secondary sexual characteristics such as breast development before 8 years of age in females (B2 according to Tanner classification) and the increase of testicular volume before 9 years of age in boys. Despite efforts to establish the genetic mechanism underlying normal and precocious pubertal timing in humans, it remains largely unknown [2]. However, gain-of-function mutations in the *KISS1* and *KISSR1* (previously named *GPR54*) genes and loss-of-function mutations in the makorin ring finger protein 3 (*MKRN3*) gene were shown to lead to CPP [3–6].

Kisspeptin, the peptide product of the *KISS1* gene, and its receptor, the G-protein 54 (*GPR54*) signaling complex, are essential gatekeepers of pubertal activation of GnRH neurons [2, 7–11]. An increase in kisspeptin signaling, caused by enhanced expression of *KISS1* and *GPR54* genes at onset of puberty, contributes to the activation of the gonadotropic axis. Therefore, gain-of-function mutations in *KISS1* or *KISSR1* genes may induce precocious activation of puberty.

On the other hand, Abreu et al. [6], using a whole exome sequencing approach, detected deleterious mutations in the gene encoding the makorin ring finger protein 3, which act by inhibiting factors that stimulate pubertal pulsatile GnRH secretion [12].

Since 2014, the role of KISS1, KISS1R and MKNR3 in precocious puberty has been recognized [13]. In this study, we analyzed the DNA sequence of these genes in a number of girls affected by CPP. Furthermore, we investigated the presence of SNPs in the same genes of another group of girls affected by “early” puberty (EP), which is defined as the onset of puberty between 8 and 10 years of age in girls showing breast development before the mean age of normal females. We purposed to verify a possible role of KISS1R and MKRN3 on pubertal timing in these subjects too.

## Methods

### Subjects

We enrolled 19 female subjects in our study: 13 cases with CPP and 6 with EP. At clinical examination, the patients diagnosed with CPP had to meet the following criteria: above-average height, increased growth rate and advanced bone age (evaluated using Greulich and Pyle Atlas); CPP was confirmed by pelvic ultrasound showing a longitudinal diameter of the uterus of over 3.6 mm and a high ovarian volume (> 2 ml), and serum LH peak above 5 IU/L after GnRH stimulation and pituitary MRI scan, in girls under 8 years of age. In girls with anticipated puberty, the same clinical and laboratory features occur between 8 and 10 years of age.

In the girls with early puberty, the onset of pubertal development started between 8 and 10 years of age and rapidly progressed, like in females with precocious puberty. Patients’ clinical and hormonal data are shown in Table 1.

**Table 1:** Clinical and hormonal features of patients

*Patient number*	*CCP/early puberty (EP) classificati on*	*Initial clinical manifestati* *on (Age, y)*	*Breast Tanner stage*	*Uterus length*	*BMI SDS*	*BA y*	*LH after GnRH IU/L* *FSH after* *GnRH IU/L*		*E2* *Pg/ml*	*Mother’s height* *(cm)*	*Father’s Height* *(cm)*	*Mother’s menarche* *(age, y)*	*Mutation* *GPR54*	*Mutation* *MKRN3*
*1*	EP	8.5	2	3.8	−1.02	9.5	3,1	12.3	26	154.5	172.2	10	rs 350,132	
*2*	EP	8.5	2	3.5	1.52	9	9,3	15.1	27.4	153		11		rs2239669
*3*	EP	8.5	3	4.3	0.26	10.5	10.74	8.4	22.1	157.1	171	11	rs 350,132	rs2239669
*4*	EP	8.5	2		−0.24		7.1	11.5	33.8				rs 350,132	
*5*	EP	8.5	2	4.6	−1.2	8.5	42.4	12.4	18	156	160.6	12	rs 350,132	
*6*	EP	8.5	2		−0.79	12	4.7	6.6	10					rs2239669
*7*	CCP	7	3	4.5	−1.1	11	35.8	17.6	< 10	158	164	11	rs 350,132	
*8*	CCP	8	3	4.2	−0.9	10.5	9.3	8.8	50.5	158.1	179.9	10	rs 350,132	
*9*	CCP	8	2	4	−1.96	8.5	5.4	7.1	20.7	168.5	173.1	13	rs 350,132	rs140467331
*10*	CCP	6.5	2	4.2	0.15	8	4.4	15.4	27.7	162.5	175.5	11	rs 350,132	
*11*	CCP	8	2	3.6	0.08	10	10.3	11	28.5	172.2	178.9	13	rs 350,132	rs2239669
*12*	CCP	7.5	2	5.2	0.12	10	15.7	9.9	19.2	163.9	169.1	14	rs 350,132	
*13*	CCP	7	3	3.5	−0.12	10.5	22.3	11.6	15.7	168	187	11		rs2239669
*14*	CCP	8	2	3.1	−0.47	11	5.7	11.2	< 10	146.8	175.4	11	rs 350,132	
*15*	CCP	8	2	5.4	0.62		36.3	13.6	38.6				rs 350,132 /rs764046537	
*16*	CCP	8	3	4.8	−0.16	10	8.1	14.8	13.5				/rs764046537	
*17*	CCP	8	3	3.6	−0.22	10.5	28.8	13.4	18				rs 350,132	rs2239669
*18*	CCP	7.5				11							rs 350,132	rs760981395
*19*	CCP	5.5	2	4.5		6	5.9	25.2	15.2				rs 350,132	

Family history of pubertal precocity was investigated and both parental height and pubertal timing were obtained.

### Hormone assays

Serum levels of LH (luteinizing hormone), FSH (follicle stimulating hormone), and estradiol (E_2_) were determined by chemiluminescent immunometric assay (Siemens Medical Solutions Diagnostics, Milan, Italy).

For the GnRH stimulation test, after administration of 100 μg of synthetic GnRH (100 mcg/m^2^) by intravenous bolus, serial blood samples for LH and FSH measurements were collected at 0, 15, 30 and 90 min after GnRH administration. LH assays had a detection limit of 0.1 IU/l. We considered stimulated LH levels of more than 5 IU/l as a pubertal cut-off.

### Genetic analysis

Genomic DNA was isolated from peripheral blood mononuclear cells (PBMC) of all patients using the Maxwell 16 Instruments (Promega). All coding exons (exon 1 to 5) and intronic flanking regions of the *KISS1R (GPR54)* gene were PCR amplified with five specific pairs of primers as previously described [7]. The entire coding region of *MKRN3* (GenBank accession number NC_000015.1) was amplified by PCR using three pairs of primers as described by Abreu et al. [6]. To confirm the presence of the rs760981395variant in our patients, we used another homemade reverse primer designed with Primer3 software. PCR products were visualized on 1.5% agarose gel, stained with ethidium bromide in order to verify the presence of PCR products. Then, DNA sequencing reactions were conducted using the same primer pairs and a BigDye Terminator V3.1 Cycle Sequencing Kit (Applied Biosystems, Foster City, CA, USA) in accordance with the manufacturer’s instructions. The sequencing reactions were electrophoresed and analyzed using the ABI PRISM 310 genetic analyzer (Applied Biosystem).

## Results

### Clinical features

Patients with CPP and EP had typical clinical and hormonal features of premature activation of the reproductive axis, including early pubertal signs such as breast development, increased growth velocity, advanced bone age, enlarged ovary volume and longitudinal uterine length, elevated E2 levels and elevated GnRH stimulated LH and FSH levels. Detailed clinical parameters are shown in Table 1. The average age at onset of symptoms was 7.46 ± 0.22 years for CPP patients and 8.5 for EP patients (all of whom were 8.5 years old). The median age plus and minus SDS at the time of diagnosis was 8.3 ± 0.36 years and 9.2 ± 0.18 years respectively; the median bone age was10.25 years (range: 9.25–10.75 y) and 9.5 years (range: 8.87–10.87 y), respectively. The median GnRH stimulated LH level was 9.8 IU/L (range: 5.8–25.5 IU/L) and 8.2 IU/L (range: 4.7–10.74 IU/L), respectively, while the median GnRH stimulated FSH level was 12.5 IU/L (range: 10.45–15.1 IU/L) and 11.9 IU/L (range: 8.4–12.4 IU/L), respectively. The median E2 level was 19.95 pg/ml (range: 15.7–28.5 pg/ml) and 24 pg/ml (range: 18–27.4 pg/ml), respectively. Patients were treated with a depot GnRH agonist resulting in adequate clinical and hormonal regression of pubertal signs and inhibition of auxological progression.

### GPR54 and MKRN3 genes analysis

Direct sequencing of the *KISS1R (GPR54)* gene revealed two SNPs (Table 2). One SNP is a missense variant (rs350132) that has been previously reported in connection to CPP in Korean girls [14] as well as in the study of another population [15]. We found this SNP in 15/19 (79%) of our patients (10 were homozygous and 5 heterozygous; in 11/13 CPP patients and 4/6 EP patients). The other variant that we found in the *GPR54* gene (rs764046557) was a missense SNP located in exon 5 at position 209 of the aminoacid. We identified this variant in only one CPP patient. This patient was also homozygous for the rs350132 polymorphism. The variant (rs764046557) is known and included in the SNP data base (TOPMed Whole Genome Sequencing (WGS) Project) with a frequency of T = 0.00002.

**Table 2:** Mutation position and characteristics: the positions of polymorphisms and mutations are defined according to contig NC_000015.10 for *MKN3* gene and NC_000019.10 for *GPR54* gene

*N*	*Gene*	*Position*	*Allele*	*Location*	*dbSNP ID*	*N patient*	*Note*
*1*	GPR54	chr19:920642	T > A T > C	Exon 5	rs 350,132	15	Benign polymorphism
*2*	GPR54	chr19:920421	C > T	Exon 5	rs764046537	1	Missense variant
*3*	MKRN3	chr15:23566445	C > A C > T	–	rs2239669	6	Synonymous variant
*4*	MKRN3	chr15:23566883	C > T	–	rs140467331	1	Synonymous variant
*5*	MKRN3	chr15:23566885	C > G	–	rs760981395	1	Missense variant

Automatic sequencing of *MKRN3* in all patients revealed three variants in eight subjects. In 6 out of 19 (31.5%) patients (3/13 CPP patients and 3/6 EP patients) we found the synonymous variant c.663C > T (rs2239669). According to ExAC, the allele frequency of this polymorphism is 0.2815 [16].

Another synonymous variant (rs140467331) was found in one of our CPP patients, as well as one missense variant (rs760981395) in another CPP patient. These variants are known and included in the SNP data base (ExAC) with a frequency of 0.00002 and 0.00001, respectively but, to the best of our knowledge, they have not yet been described in scientific papers in connection to CPP. The missense variant caused an aminoacid substitution (Ser to Cys) at position 368.

## Discussion

In pediatric endocrinology practice, precocious puberty is a non-rare condition that can result in short final height and physiological complaints in untreated patients [17]. Increased evidence suggests an association between early timing of puberty and adverse health outcomes in later life such as risk of breast neoplasia.

The onset of puberty varies greatly among individuals and races, and much of this variation is due to genetic factors. Kisspeptin and its receptor, GPR54, appear to be the crucial regulators of puberty [1]. Since 2003, many researchers have attempted to find a molecular mechanism of the kisspeptin/KISS1R system that can be associated with puberty development variation. They found mutations in the *GPR54* gene that resulted in idiopathic hypogonadotropic hypogonadism [1, 11, 18, 19].

However, the genetic basis of CPP includes either mutations in the *KISS1* and *KISS1R* genes or loss-of-function mutations in the *MKRN3* gene. To date, *MKRN3* mutations have been described in 58 patients with CPP from 35 different families [2, 6, 12, 20–24]; these include 23 different loss-of-function and 11 missense mutations of *MKRN3 *and represent the most frequent genetic cause of CPP since being identified in 2013 [10]. Makorin ring finger protein 3 is codified by an intron less gene on chromosome 15 and is thought to have an inhibitory effect on GnRH secretion. It belongs to a family of E3 ubiquit in ligases, but its mechanism of action is as yet unknown.

In 2008, an autosomal dominant missense mutation in KISS1R, leading to prolonged activation of intracellular pathways in response to kisspeptin, was suggested as a cause of CPP [4]. Since then, no other CPP cases with activating *KISS1R* mutations have been reported, but a few *KISS1R* polymorphisms have been identified in CPP patients [13, 25]. In this study, we detected one SNP missense variant (rs 350132c.1091 T > A), which had been previously reported in connection to CPP in Korean girls [14] as well as in the study of another population [15]. It is a non-synonymous SNP that induces amino acid substitution of p.Leu364His, and it was considered as a benign polymorphism in three in silico analyses used by Yean Joung Oh et al. [25]. Interestingly, we found this SNP in both groups of patients in our study: CPP and EP patients. Therefore, we can suppose that this variant may also be associated with moderately early onset of puberty. The other variant that we found in the *GPR54* gene (rs764046557) was a missense SNP, located in exon 5 of the aminoacid at position 209. We identified this variant in one CCP patient only. These patients were also homozygous for rs 350,132 polymorphism. This variant is known and included in the SNP database (TOPMed Whole Genome Sequencing (WGS) Project) but it has not been previously described in scientific papers as being linked to CPP or to any pathological effect.

Analysis of the *MKRN3* gene in all our patients revealed three variants in eight subjects. In 6 out of 19 (31.5%) patients (3/13 CPP patients and 3/6 early puberty patients) we found the synonymous variant c.663C > T. Additive model analysis revealed a significant link between this SNP and precocious puberty in boys but, in contrast, no association was found in CPP girls [26]. However, Ortiz-Cabrera et al. [17] found this variant in 40% of their female patients (expected frequency 20%) and they speculated that some SNPs, although silent in relation to the protein aminoacid chain, could affect the expression of post-translational features of the gene, leading to malfunction.

We found another synonymous variant (rs140467331) and one missense variant (rs760981395) in two CPP patients. These variants are known and included in the SNP database (ExAC) but, to the best of our knowledge, they have not yet been described in scientific papers in connection to CPP. The missense variant caused an aminoacid substitution (Ser to Cys) at position 368.

There were no clinical or biological features in our patients suggestive of *MKRN3* mutations; in fact, variations were found in both groups, i.e. precocious and early puberty. This is in contrast with other studies in which the authors found a significant difference in the median age between girls with or without mutations [27]. However, it is possible that some patients presented at the hospital at a late pubertal stage and so, the time from the onset of puberty symptoms to diagnosis may not be precise, as it was estimated on data reported by parents.

Our paper confirms the presence (and expands the phenotype) of mutations and polymorphisms of *KISS1R* and *MKRN3* genes, not only in ICPP patients but also in early puberty subjects. However, our study has some limitations: we were not able to perform functional studies to demonstrate the pathogenicity of the variants we found; in addition, it was not possible to obtain DNA samples from the fathers of patients with mutations in order to verify paternal inheritance, which had been demonstrated in previous reports [16]; finally, our sample scale was quite small.

## Conclusions

We have attempted to identify sequence variations of the *KISS1R* and *MKRN3* genes, two of most frequent genetic causes of CPP, and our findings suggest that these variants might be inducible factors in the pathogenesis of CPP. Furthermore, two recent studies have revealed a negative correlation between circulating MKRN3 levels and the Tanner stages of puberty in healthy children and adolescents [27, 28]. Therefore, screening is recommended, in particular for MKRN3 mutations, for all patients with familial ICPP, patients with unclear family histories [3] and patients with sporadic CPP [2]. It is also important that the younger siblings of patients with familial CPP secondary to *MKRN3* mutations be screened for the same mutations. In fact, this approach ensures early diagnosis and prompt GnRH agonist treatment.

## Data Availability

The datasets used and/or analyzed during the current study are available from the corresponding author on request.

## Abbreviations

FSH: Follicle stimulating hormone
LH: Luteinizing hormone
GnRH: Gonadotropin releasing hormone
E_2_: Estradiol
SD: Standard deviation
SDS: Standard deviation score
MKRN3: Makorin ring finger protein 3
KISS1R: (or GPR54): Kisspeptin receptor
CPP: Central precocious puberty
EP: Early puberty
